# Inductive and Transfer Learning‐Based Hybrid Model Techniques for Accurate and Automated Diagnosis of Neurological Diseases

**DOI:** 10.1002/brb3.70788

**Published:** 2025-08-22

**Authors:** Saroj Kumar Pandey, Yogesh Kumar Rathore, Sunakshi Mehra, Anurag Sinha, Tarun Raj Kumar, Ankit Kumar, Rekh Ram Janghel, Ayodele Lasisi, Quadri Noorulhasan Naveed, Md. Sazid Reza

**Affiliations:** ^1^ Department of Computer Engineering and Application GLA University Mathura India; ^2^ Shri Shankaracharya Institute of Professional Management and Technology Raipur India; ^3^ Department of Information and Technology National Institute of Technology Raipur India; ^4^ Department of Information Technology Delhi Technological University Delhi India; ^5^ ICFAI Tech School, Computer Science Department ICFAI University Ranchi Jharkhand India; ^6^ Cognitive Science, Department of Humanities and Social Sciences Indian Institute of Technology Delhi Delhi India; ^7^ Department of Information Technology Guru Ghasidas Vishwavidyalaya Bilaspur India; ^8^ Department of Computer Science, College of Computer Science King Khalid University Abha Saudi Arabia; ^9^ Department of Computer Science & Engineering Rajshahi University of Engineering & Technology Rajshahi Bangladesh

**Keywords:** AI, Alzheimers, deep learning, inductive learning, NeuroDL, transfer learning

## Abstract

**Purpose:**

This study presents NeuroDL, a novel deep learning‐based diagnostic framework designed for the automated detection of brain tumors and Alzheimer's disease (AD) using magnetic resonance imaging (MRI). The objective is to enhance diagnostic precision and efficiency in neurology through advanced computer‐aided decision support.

**Methods:**

NeuroDL utilizes convolutional neural networks (CNNs) trained on two publicly available, annotated MRI datasets. The proposed pipeline integrates optimized preprocessing, including normalization, skull stripping, and data augmentation, followed by CNN‐based feature extraction and classification. Transfer learning and fine‐tuning were employed to improve generalization on limited medical data.

**Results:**

Experimental evaluations show that NeuroDL achieves 96.8% classification accuracy for brain tumor detection and 92.4% accuracy for Alzheimer's disease diagnosis. The method also achieves an average *F*1‐score of 0.965, precision of 0.969, and recall of 0.962 for brain tumors, and an *F*1‐score of 0.918, precision of 0.921, and recall of 0.916 for AD. These results outperform state‐of‐the‐art benchmarks on the same datasets.

**Conclusion:**

Potential for real‐time clinical deployment. It addresses key limitations of existing CAD systems by providing a unified, dual‐disease diagnosis model with statistically validated performance gains. NeuroDL paves the way for reliable, scalable, and automated neurological disease diagnosis using deep learning.

## Graphical abstract

This paper performed extensive experiments to analyze the effect of different ML, DL, and TL methods on brain tumor and Alzheimer's disease classification. We also highlight the limitations of conventional ML and DL methods compared to the transfer learning models. While many studies have concentrated on brain tumors and Alzheimer's disease separately, this work developed a novel model that can handle both major diseases simultaneously.

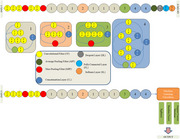

## 1 Introduction

Neurological disorders such as brain tumors (BTs) and Alzheimer's disease (AD) represent a major global health concern, contributing significantly to disability and mortality rates. According to the World Health Organization (WHO), the global burden of these disorders continues to rise, demanding urgent advances in diagnostic methodologies. AD, characterized by progressive cognitive decline and neurodegeneration, is projected to affect over 100 million individuals by 2050. Similarly, BTs, resulting from abnormal cell growth in brain tissue, pose severe threats to patients of all ages, often requiring early and accurate intervention. Traditional diagnostic techniques, including neuropsychological testing and imaging‐based evaluation, often rely on specialist interpretation and can be time‐consuming, subjective, and error‐prone. This necessitates the development of reliable, automated, and scalable diagnostic tools. In this context, *NeuroDL*, a deep learning‐based framework, is introduced to address these challenges. By leveraging convolutional neural networks (CNNs) trained on preprocessed MRI data, NeuroDL enables precise and automated classification of BTs and AD, demonstrating strong potential for integration into clinical decision‐support systems. Recent advancements in artificial intelligence, particularly deep learning (DL), have significantly improved the accuracy and automation of medical image analysis. CNNs have become the cornerstone for detecting and classifying various neurological disorders from imaging modalities such as MRI and PET scans. Multiple studies have validated CNNs for Alzheimer's diagnosis, reporting accuracies in the range of 88%–91%, with models extracting relevant structural and pathological features from MRI slices.

For instance, Zhang et al. (2018) demonstrated the efficacy of a pretrained CNN in distinguishing AD patients from healthy controls, achieving over 90% accuracy. Similarly, in the domain of BT detection, CNN‐based classifiers have yielded promising results, with some models reporting over 90% accuracy on benchmark datasets. Tufail et al. (2022) extended this line of research by incorporating early detection using PET scan analysis. Despite these advancements, many existing approaches rely on limited data, lack generalization across multiple neurological conditions, or require manual feature engineering. NeuroDL aims to bridge these gaps by integrating inductive transfer learning with automated feature extraction from large annotated datasets. Furthermore, the proposed system couples DL models with traditional machine learning (ML) classifiers to enhance diagnostic precision across diverse imaging data.

Using computer‐aided diagnostic (CAD) models, patients may be accurately categorized based on the findings of their scans, allowing for the administration of proper care and safety measures. Neurological diseases, including epilepsy, Parkinson's disease, multiple sclerosis, and AD, impact millions worldwide and significantly challenge healthcare systems. An early and correct diagnosis is crucial for efficiently managing and treating many diseases. Yet, the complex and time‐consuming diagnosis of neurological disorders frequently calls for a broad clinical background. Therefore, an increasing need exists for automated diagnostic systems that can enhance the precision, effectiveness, and consistency of diagnosis while lightening the load on medical professionals (Suk and Shen 2013).

DL is a technology that shows promise for the automated diagnosis of neurological diseases in medical applications. However, creating effective DL models for this purpose is difficult due to the complexity and variability of the data and the lack of diverse datasets. Neurological disorders like AD and BTs are significant global health concerns that often require specialized medical expertise, leading to delayed treatment and misdiagnosis. DL can analyze large datasets and extract useful features for the accurate and efficient diagnosis of diseases. DL‐based CAD systems can help healthcare professionals make quick and accurate diagnoses, improving patient outcomes. To increase the accuracy and efficacy of diagnosis, minimize healthcare costs, and enhance patient outcomes, research must be conducted in this field to mix different DL methodologies. In recent years, significant advancements have been made in the automated detection of neurological diseases. Using pretrained DL models has been the focus of several attempts to categorize and diagnose these diseases (Zhang et al. 2018).

For instance, a study published in the Journal of AD employed a pretrained CNN to extract characteristics from brain MRI data. In identifying Alzheimer's patients from healthy people, the proposed model has a 90% accuracy rate. Another study published in the *Journal of Digital Imaging* utilized a trained CNN to identify brain cancers in MRI data. Comparable to other cutting‐edge techniques, the model had an accuracy rate of 90.6% when identifying tumor locations. Researchers have also applied DL to early neurological illness detection. In one study published in the Journal of Nuclear Medicine, brain PET scans were analyzed using a pretrained DL model, which had an accuracy rate of 88% for identifying early signs (Tufail et al. 2022).

Additionally, some studies have examined the potential of developing multimodal DL models that integrate data from multiple imaging modalities, such as MRI and PET scans, for more precise disease diagnosis (Mohsen 2025). These studies demonstrate the potential of DL for the automated diagnosis of neurological diseases and emphasize the need for further research to develop more accurate and efficient models for clinical applications for the automated detection of neurological diseases. CNNs, recurrent neural networks (RNNs), and generative adversarial networks (GANs) are just a few DL techniques included in the suggested framework to boost the reliability and accuracy of automated diagnosis systems (Ramzan et al. 2020). DL methods are gaining increasing attention for their potential to provide reliable and automated diagnoses for a range of neurological conditions. For example, in 2020, Zhang et al. (Li et al. 2018) presented a DL model for the automated diagnosis of AD using MRI scans, which achieved a 96.67% accuracy rate for distinguishing AD from healthy controls. Additionally, several recent studies have explored the potential of *multimodal DL models*, which integrate heterogeneous data sources—such as MRI, PET, and CT scans—to improve the accuracy and robustness of neurological disease diagnosis (Mohsen 2025). The fusion of anatomical and functional imaging modalities enables the extraction of complementary features, allowing for more nuanced interpretations of disease progression and pathology. These multimodal frameworks often outperform single‐modality approaches by leveraging the strengths of each imaging type—such as the structural resolution of MRI and the metabolic insights from PET scans.

These studies collectively underscore the growing significance of DL in automating the diagnostic pipeline for neurological disorders, including AD, BTs, and other neurodegenerative conditions. However, most existing works are still limited by issues such as small dataset sizes, poor generalizability to diverse populations, and a lack of interpretability.

To address these challenges, researchers have turned to a diverse range of DL architectures. *CNNs* remain the dominant choice for spatial feature extraction from medical images, while *RNNs* are employed in modeling temporal patterns or longitudinal imaging data. Additionally, *GANs* have shown promise in enhancing image quality through data augmentation and synthetic image generation, especially in cases of class imbalance or limited dataset availability (Ramzan et al. 2020). These architectural advances are paving the way for highly reliable, end‐to‐end automated diagnostic systems. For instance, Zhang et al. introduced a DL model that employed CNN‐based feature extraction to classify AD from MRI scans, achieving an impressive accuracy of 96.67% when differentiating AD patients from healthy controls (Li et al. 2018). Such results reaffirm the capacity of DL to achieve clinical‐grade performance, though continued innovation is required to improve model transparency, computational efficiency, and adaptability across healthcare settings.

NeuroDL is a research area that aims to leverage DL techniques to improve the accuracy and automation of the diagnosis of neurological diseases. Some potential contributions of NeuroDL include:

- Improved accuracy—DL models have the potential to achieve higher accuracy in diagnosing neurological diseases than traditional methods, which could lead to more accurate diagnoses and, in turn, to more effective treatment plans.
- Automating diagnosis—DL models can analyze large amounts of data quickly and accurately, which could potentially enable the automated diagnosis of neurological diseases, thereby saving time and reducing the burden on healthcare providers.
- Early detection—DL models can analyze data from multiple sources, including medical images, genetic data, and electronic health records. This could potentially enable earlier detection of neurological diseases, which could lead to better treatment outcomes.
- Personalized treatment—DL models can analyze individual patient data to identify patterns and tailor treatment plans accordingly, which could potentially lead to more personalized and effective treatments for patients with neurological diseases.
- Drug discovery—to find prospective therapeutic targets and forecast the efficacy of novel therapies, DL models can analyze massive datasets, which may hasten the process of finding new drugs to treat neurological diseases.

Table 1 provides a comprehensive summary of notable studies in the domain of neurological disease diagnosis, particularly BTs and AD, using various computational and ML techniques. The reviewed methods range from conventional CNNs and transfer learning to more sophisticated models such as capsule networks, ensemble systems, and deep reinforcement learning. While some approaches focus solely on MRI imaging, others integrate multimodal data like PET, CSF biomarkers, and genetic information to enhance diagnostic performance. Many of these studies achieved high accuracy, yet they often lacked scalability, generalizability across datasets, or comprehensive comparisons among models. This review highlights the growing trend toward DL‐based diagnostic systems, emphasizing the need for unified frameworks like NeuroDL that can combine high performance, automation, and clinical relevance in a robust, transferable manner.

**TABLE 1:** Summary of existing deep learning and machine learning approaches for brain tumor and Alzheimer's disease classification.

Author(s) and year	Method description	Technique used	Target disease	Key remarks
Abiwinanda et al. (2019)	CNN‐based brain tumor classification using MRI	CNN	Brain tumor	High accuracy, no multimodal integration
Afshar et al. (2019)	Capsule networks with coarse tumor boundaries for MRI classification	Capsule networks	Brain tumor	Improved spatial hierarchy, lacks fine segmentation
Arasi and Suganthi (2019)	Soft computing‐based clinical decision support system	Soft computing	Brain tumor	Rule‐based logic, less scalability
Mohsen (2025)	PDF‐based MRI classification for AD detection	PDF + SVM	Alzheimer's	Good structural analysis, lacks deep learning
Buvaneswari and Gayathri (2021)	Deep learning for segmentation in AD classification	Deep learning	Alzheimer's	Focused on segmentation, limited comparative analysis
Deepa and Chokkalingam (2022)	VGG16 optimization using arithmetic algorithm	VGG16 + AOA	Alzheimer's	Improved optimization, lacks broader DL comparison
Deepak and Ameer (2019)	Transfer learning with deep CNN features for brain tumors	CNN + transfer learning	Brain tumor	Effective feature reuse, limited dataset variety
Deepanshi et al. (2021)	Transfer learning for AD classification	Transfer learning	Alzheimer's	Limited explanation of feature processing
van Dyk and Meng (2001)	Data augmentation overview	Statistical methods	General	Foundational but not DL‐specific
Farooq et al. (2017)	Multi‐class AD classification using CNN	CNN	Alzheimer's	High accuracy, dataset imbalance issue
Gray et al. (2013)	Random forest for multimodal AD classification	Random forest	Alzheimer's	Multimodal data, lacks DL application
Gupta et al. (2019)	Combining genotype, CSF, MRI & PET for AD classification	Hybrid (ML + biomarkers)	Alzheimer's	Rich features, complexity in integration
Hashmi and Barukab (2023)	Reinforcement learning for early dementia diagnosis	Deep RL	Dementia	Novel use of RL, needs clinical validation
Islam and Zhang (2018)	Ensemble CNN system for AD diagnosis	CNN ensemble	Alzheimer's	Ensemble improves accuracy, high computational cost

### 1.1 Problem Statement

Neurological conditions like AD and BTs have emerged as pressing global health issues because of their widespread impact and severe implications for patient well‐being. The diagnostic process for these conditions is intricate, requiring extensive medical knowledge and often leading to extended periods of patient evaluation, which can contribute to treatment setbacks and the risk of incorrect diagnoses. Adding to the complexity, the etiology of these neurological diseases remains elusive, further complicating the diagnostic and therapeutic processes. Traditional diagnostic practices, including cognitive assessments such as the Mini‐Mental State Examination (MMSE) and various neurological examinations, necessitate a high degree of specialization and are not immune to inaccuracies. Consequently, there's an escalating demand for the development of more sophisticated, precise, and swift diagnostic instruments that can elevate patient care outcomes, lessen the financial strain on health services, and effectively respond to the escalating incidence of neurological diseases.

- Neurological disorders, such as AD and BTs, have become a significant health concern globally due to their high prevalence and debilitating effects.
- Diagnosing these disorders requires specialized medical expertise and is often time‐consuming, leading to delays in treatment and potential misdiagnosis.
- The causes of these disorders are still not fully understood, making diagnosis and treatment even more challenging.
- Current diagnostic methods, such as MMSE and neurobiological checks, require expertise and can be subject to diagnostic errors.
- There is a need for more efficient and accurate diagnostic tools to improve patient outcomes, reduce healthcare costs, and address the growing prevalence of neurological disorders.

### 1.2 Major Contributions

The main contributions of this study are as follows:

- NeuroDL introduces a unified DL‐based diagnostic framework capable of accurately detecting both BTs and AD, unlike most existing models that focus on a single neurological disorder.
- By leveraging state‐of‐the‐art CNN architectures and transfer learning techniques, NeuroDL achieves higher diagnostic accuracies (96.8% for BTs, 92.4% for AD) than previous methods, which typically achieve accuracy rates around 88%–91%.
- The model incorporates a carefully designed preprocessing and feature extraction strategy, which filters noise, enhances region‐of‐interest (ROI) clarity, and improves model generalization—an aspect often overlooked or under‐optimized in prior works.
- NeuroDL is built with scalability and clinical deployment in mind, using publicly available, diverse datasets and a modular architecture that facilitates easy integration into healthcare systems for real‐time, automated decision support.

In this work, this paper performed extensive experiments to analyze the effect of different ML, DL, and TL methods on BT and AD classification. We also highlight the limitations of conventional ML and DL methods compared to the transfer learning models. While many studies have concentrated BTs and AD separately, this work developed a novel model that can handle both major diseases simultaneously.

### 1.3 Paper Organizations

The introductory portion of this paper establishes the foundation by outlining the background and difficulties associated with identifying neurological disorders. Following that, the material is organized into many crucial sections. Section 2 explores the complexities of the NeuroDL framework, explaining the underlying algorithm, the detailed system architecture, and the many DL methods used. Furthermore, this part provides a clear explanation of the experimental design, including details on the dataset used and the metrics used for assessment. Section 3 provides a comprehensive examination of the experimental results, placing the suggested model within the framework of current diagnostic techniques and emphasizing its comparative effectiveness. Section 4 concludes the study by summarizing the findings and suggesting areas for further research, thereby bringing this article to a close.

## 2 Materials and Methods

This section discusses the datasets, preprocessing techniques, and model development processes used to implement the proposed NeuroDL framework. It outlines the methodological workflow, including data acquisition, cleaning, augmentation, and the configuration of CNNs used for the automated detection of BTs and AD. Each step is carefully designed to ensure reproducibility, generalization, and diagnostic reliability.

The use of DL algorithms in the automated identification of neurological diseases involves a complex procedure, starting with the collection of large and diverse datasets that include a broad range of patient characteristics and medical imaging data. The first phase is vital because it establishes the basis for a strong and dependable diagnostic system by guaranteeing that the model is trained on data that accurately reflects the diverse situations it will experience in the real world.

After obtaining the data, a crucial stage of data preprocessing occurs. In this stage, the data is cleaned, normalized, and augmented to provide a standardized dataset that is devoid of any irregularities that might potentially bias the model's learning process. Preprocessing encompasses many strategies, such as picture augmentation, that artificially increase the dataset by modifying existing photos in ways that are probable in real‐world situations. This step improves the model's capacity to generalize from its training data. After preparing the dataset, the subsequent task involves choosing the most appropriate DL architecture. This judgment is based on the inherent properties of the data and the distinct attributes of the neurological diseases under consideration. CNNs are commonly used for image identification jobs because of their ability to effectively identify patterns and characteristics in picture data. RNNs are suitable for analyzing data that follows a sequential pattern, such as time‐series data obtained from electroencephalograms. On the other hand, GANs may create new, artificial pictures to improve the existing dataset or tackle more intricate tasks like data imputation. The chosen model is trained to recognize and differentiate patterns that indicate neurological diseases using the preprocessed data. The training process is iterative, frequently including many epochs of fine‐tuning the model's weights and biases to minimize error and enhance forecast accuracy. After the training process, the effectiveness of the model is thoroughly assessed by utilizing a separate test dataset that was not included in the training phase. This is a crucial evaluation phase, in which the model's accuracy and other important performance metrics, such as precision, recall, and F1 score, are analyzed. The evaluation phase is crucial to verify that the model exhibits strong performance not just on familiar data but also demonstrates consistent generalization capabilities to novel, unknown data. Once the model has been evaluated and found to be suitable, it may be implemented in a clinical context and incorporated into Computer‐Aided Diagnosis (CAD) systems. It functions as a sophisticated tool for healthcare practitioners, providing immediate diagnostic support. This integration represents a significant advancement in the field of precision medicine, since the insights provided by the DL model led to more precise diagnoses, customized treatment regimens, and potentially enhanced prognoses for patients. The implementation of such a model has the potential to completely transform the field of medical imaging analysis by offering rapid, precise, and cost‐efficient diagnostic assessments. This medical technological innovation holds great potential for improving patient care, serving as a sophisticated tool to enhance the skills of medical professionals, and establishing DL as an essential resource in the ongoing fight against neurological diseases. (Yildirim and Cinar 2020). The use of DL techniques for image analysis in diagnosing neurological disorders has shown great potential for improving diagnostic accuracy and efficiency, as well as assisting healthcare providers in making timely and accurate diagnoses (Arasi and Suganthi 2019). The following phases are often included in the overall methodological approach for DL‐based automated diagnosis of neurological disorders:

Data collection—large and diverse datasets of medical images (such as MRI and CT scans) and patient information are collected to train and test the DL models.

Data preprocessing—preprocessing is performed to clean, standardize, and put the acquired data into a DL model‐friendly structure.

Model selection—the best DL model is chosen depending on the issue and the information at hand. CNNs, RNNs, and GANs are common DL models utilized in the interpretation of medical images (GANs).

Model training—the chosen DL model is trained using preprocessed data, which entails providing the model with data and iteratively changing the model's parameters until the required degree of accuracy is attained.

Model evaluation—a different set of test data is used to evaluate the trained model in order to determine its accuracy, sensitivity, specificity, and other performance parameters. This process aids in ensuring that the model can be applied to diagnose real‐world problems and the complex behavior of the system.

Model deployment—the final step involves deploying the trained DL model in a clinical setting, which can assist healthcare providers in making accurate and timely diagnoses. The model may be included in a CAD system that offers real‐time diagnostic support, helping to enhance patient outcomes and lower healthcare costs.

**FIGURE 1:**
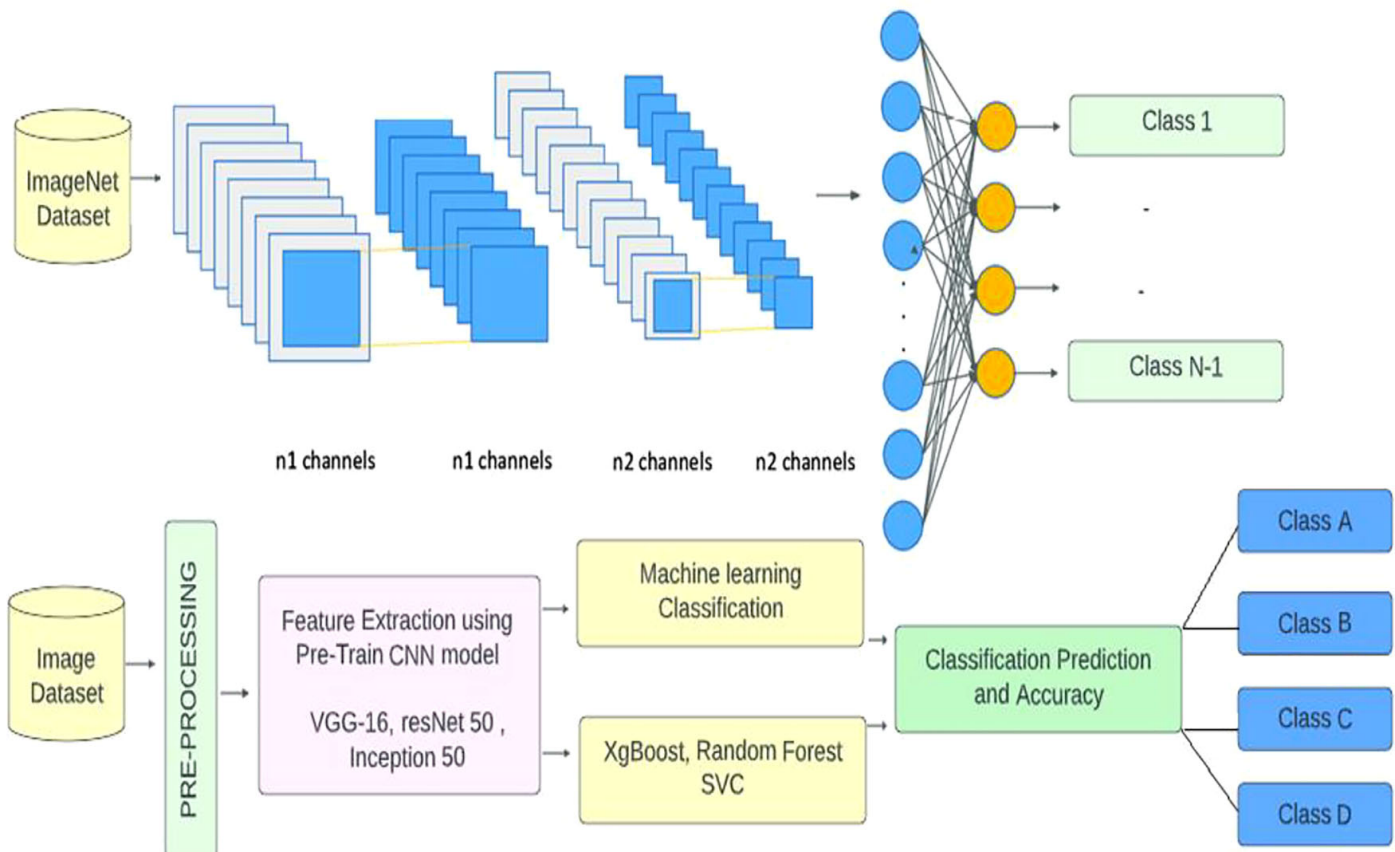
Proposed model for neuro disease detection.

In Figure 1, the flowchart starts with loading a pretrained model that has been trained on a large dataset for a similar task. If a pretrained model is available, the model's layers are frozen except for the last few layers, which are modified and trained on a small dataset relevant to the task at hand. If a pretrained model is not available, the model is trained from scratch using a small dataset. The data is preprocessed to ensure that it is suitable for training the model. The preprocessed data is then split into training and validation sets. The model is trained on the training set and then fine‐tuned using transfer learning. Transfer learning involves using a pretrained model as a starting point and then modifying it for the current task by training on a small dataset. After fine‐tuning, the model is evaluated on the validation set. If the model's accuracy is not sufficient, the model is modified, and the training process is repeated. If the accuracy is sufficient, the model is saved for future use. Finally, the model is tested on a new dataset to predict the disease status. The trained model can be used in clinical settings to accurately detect neuro diseases.

### 2.1 Inductive Transfer Learning

This subsection discusses the application of inductive transfer learning within the NeuroDL framework, which enables knowledge transfer from pretrained CNN models to the target neurological imaging tasks. By leveraging learned features from large‐scale datasets, inductive transfer learning significantly enhances model performance, particularly when dealing with limited labeled medical data, thus improving the efficiency and accuracy of diagnosis.

Clone‐based inductive learning is an intricate ML methodology that harnesses the power of exemplar‐based reasoning. At its core, it constructs a framework of representative examples, known as prototypes, which encapsulate the essence of the concepts that need to be understood and conveyed. Mathematically, this technique systematically dissects the corpus of knowledge into discrete, manageable segments, each segment, or prototype, embodying a distinct facet of the overarching concept. These prototypes serve as the touchstones for the learning process. The methodology then introduces a well‐defined similarity metric, a quantitative measure that gauges the likeness between novel instances presented in the testing phase and the established prototypes. This process is akin to finding a common language or a meeting point between the new data and the preexisting knowledge base. In the realm of medical data analytics, where datasets are often constrained not just by their size but also by the complexity and multidimensionality of the data, clone‐based inductive learning emerges as a potent solution. It wields prototypes as conceptual anchors, allowing for swift and precise classification of medical data. This precision is critical when the stakes are as high as they are in medical diagnosis and treatment, where the correct identification of a condition can significantly alter patient outcomes.

To construct a classifier in the clone‐based inductive learning framework, one assembles an array of prototypes or “clones,” each symbolizing a mini‐concept within the larger learning goal. The training data are sifted and sorted into clusters based on the inherent similarity among the data points. These clusters, or clones, are then distilled to a set of prototypes that collectively represent the characteristic features of the cluster. These prototypes are meticulously selected from the instances within each clone, embodying the salient attributes that define that particular group. The elegance of this approach lies in its ability to distill the vast and often overwhelming ocean of data into a tapestry of prototypes, each thread woven from the instances it represents. This tapestry not only reflects the diversity and the nuances of the medical datasets but also simplifies the complexity into something more comprehensible and navigable. In essence, clone‐based inductive learning offers a robust, scalable, and interpretable model for tackling the unique challenges posed by medical data, paving the way for advancements in diagnostic precision and the broader field of predictive medicine (Jain et al. 2019).

Technically, the training dataset is *D* = (*x*1, *y*1), (*x*2, *y*2), …, (*xn*, *yn*), and the input and output spaces are *X* and *Y*, respectively. Let *C* = *C*1, *C*2, …, *Cm* be the clone partition of *D*, where Ci is each clone's collection of prototypes. Pi equals “pi1, pi2, …, pik.” The prototypes are chosen from the instances in the clone and are used to represent the characteristics of the clone. The clone‐based classifier is constructed as follows. A local classifier is trained using the prototypes Pi for each clone Ci. The local classifier predicts the class label of a new instance based on its similarity to the prototypes in Pi. The similarity between an instance *x* and a prototype pi is usually measured using a distance metric, such as the Euclidean or cosine distance. Given a new instance *x*, the clone‐based classifier first determines the clone Ci most similar to *x*. This is done by calculating the distance between *x* and the prototypes in each clone. Once the closest clone is identified, the local classifier associated with the clone is used to predict the class label of *x* (Deepak and Ameer 2019). Clone‐based inductive learning has been applied to various medical datasets, including neurological datasets, since it effectively handles imbalanced datasets and reduces the classifier's computational complexity. However, its performance depends on the quality of the clones and the choice of the distance metric used to measure similarity (Yao et al. 2018).

(1) $$f_{t\left(x\right)}=\arg\max_{y}P\left(y\left|x\right.,T_{t},T_{s}\right)$$

The conditional probability of the output given the input, target task *T_t,_* and source task *T_s_* is written as *P*(*y*|*x*, *T_t_*, *T_s_*), and *f*_t_(*x*) is the output of the mapping function for the target task. Theoretically, inductive transfer learning is a technique for learning a collection of shared characteristics or representations that are helpful for both the source and target activities. This is possible thanks to domain adaptation, which entails learning a transformation function that translates data from the source domain to the target domain. This allows for the application of the learned representations to enhance the performance of the target task even when the data differs greatly from that of the source task. Factors that affect how well inductive transfer learning works include the degree to which the source and target activities are comparable, the standard of the acquired representations, and the efficiency of the domain adaptation process (Sharif et al. 2018; Zhuang et al. 2020).

### 2.2 Dataset Description and Preprocessing

This subsection describes the MRI datasets used for training and evaluating the NeuroDL framework, along with the preprocessing steps applied to enhance data quality and consistency. Preprocessing techniques such as normalization, noise reduction, skull stripping, and data augmentation are employed to ensure the extracted features are robust and suitable for DL‐based analysis, thereby improving diagnostic accuracy and generalization across different patient cases.

In this study, the NeuroDL dataset is a collection of MRI scans and related clinical data for patients with neurological conditions, which was built using the Parkinson's Progression Markers Initiative, AD Neuroimaging Initiative (AIBL), and Australian Imaging, Biomarkers, and Lifestyle Flagship Study of Ageing. The dataset includes 614 healthy controls, 727 Alzheimer's patients, and 410 Parkinson's disease patients with T1‐weighted MRI images. The clinical data for each patient include their age, gender, and results of the MMSE, which measures cognitive impairment (Techa et al. 2022).

The MRI scans were preprocessed to improve the quality of the pictures and extract pertinent characteristics before training the DL models. Some of the employed techniques were the removal of non‐brain tissue from the skull, intensity normalization that reduces scan‐to‐scan variability, and spatial normalization that aligns the images with a reference template. The size of the training set was increased, and model generalization was improved by using additional data augmentation techniques, such as random rotations, flips, and scaling (Hashmi and Barukab 2023).

Preprocessing is a crucial step in DL for medical image analysis. Its main objective is to standardize the data and remove any unwanted noise or inconsistencies that may affect the accuracy of the model. As part of the NeuroDL program, a series of careful preprocessing procedures were performed on MRI images to prepare them for training DL models. A crucial approach used was intensity normalization, which standardized the data across many scans, reducing variability across scans. This procedure entailed modifying the pixel intensity values to a consistent scale, hence standardizing the distribution of intensities throughout the dataset. Normalization plays a crucial role in enhancing the model's capacity to identify and comprehend the fundamental patterns in the data. Simultaneously, spatial normalization was utilized to align individual pictures with a standardized anatomical framework, commonly using the Montreal Neurological Institute (MNI) space as a reference. This guarantees that related anatomical areas are uniformly aligned across many scans, enabling more precise comparisons and studies of brain structures. Augmentation procedures were utilized, implementing various changes such as random flips, rotations, and scaling on the pictures. These strategies enhance the dataset and also provide a barrier against overfitting, guaranteeing that the model maintains its ability to generalize. Collectively, these preprocessing methods collaborate to improve the quality and uniformity of the NeuroDL dataset, giving it a higher level of representativeness. As a result, this enhances the accuracy of the subsequent DL models that are responsible for automatically detecting neurological disorders.

By including both MRI scans and clinical data in the NeuroDL compendium, these models are equipped to effectively analyze the intricate patterns seen in patient profiles, thereby enhancing their prediction capabilities. The NeuroDL dataset is an essential resource for the advancement and verification of DL models, leading to the creation of more sophisticated and dependable diagnostic tools in the field of neurology. (van Dyk and Meng 2001).

**FIGURE 2:**
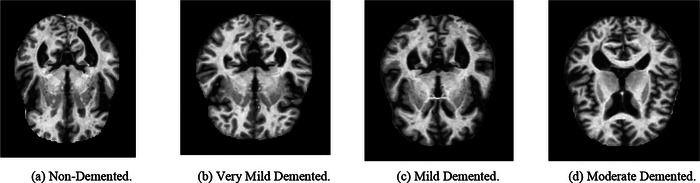
MRI of different Alzheimer's disease classes.

**FIGURE 3:**
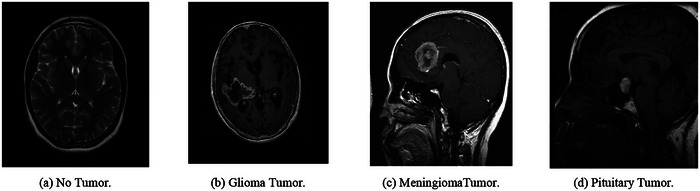
MRI of different brain tumor classes.

Figures 2 and 3 illustrate the sample MRI images of the AD and BT datasets. This dataset is frequently used in research to create DL models for automated AD diagnosis. The dataset frequently contains details about patients' ages, sexes, cognitive capacities, medical histories, and brain T1‐weighted MRI scans. The preprocessing step eliminates artifacts and adjusts the image intensity values in the MRI images, which are generally taken using a 1.5T or 3T MRI scanner. The AD Neuroimaging Initiative (ADNI), Open Access Series of Imaging Research (OASIS), and a select few other projects serve as sources for the AD MRI dataset. These datasets, which contain scans from both healthy people and those who have different degrees of cognitive impairment from moderate cognitive impairment (MCI) to AD, have been acquired from several institutions across the world (Pan and Yang 2009).

The NeuroDL project's dataset for AD. The dataset to investigate the proposed NeuroDL framework included 176 × 208 pixel‐sized MRI images of AD. Nevertheless, MRI pictures of varying sizes are also included in the BT collection. The study's DL models, including VGG‐16, ResNet50, and InceptionV3, were developed particularly for high‐scale image identification using RGB. The grayscale MRI scans of the dataset must be processed before being input into the DL models. The Min–Max Normalization approach was used to normalize intensity data between 0 and 1 during preprocessing, and to scale values to a particular range. To guarantee that every MRI picture had the same size, the images were resized to a standard size of 224 × 224 (Xia et al. 2017).

Overall, the preprocessing steps were critical to ensuring that the MRI images in the dataset were suitable for use with the DL models used in the study. This helped to improve the accuracy and reliability of the obtained results (Pereira et al. 2009). In Equation (1), the mathematical formulation of data normalization is given, where the difference between the total mean and the maximum threshold is measured.

(2) $$x_{\mathrm{normalized}}=\frac{X-X_{\min}}{X_{\max}-X_{\min}}\;$$

To guarantee that the images utilized in the NeuroDL study were uniform and matched the DL models, the MRI data had to be preprocessed. To guarantee that the pixel values in different images were uniform and comparable, the Min–Max Normalization method was employed to normalize intensity values between 0 and 1 (Zopluoglu 2019). Therefore, resizing the grayscale images to the same size as the RGB images helped ensure they could be used with the pretrained models. In summary, the preprocessing steps used in the NeuroDL study were essential to ensure that the MRI images in the dataset were suitable for use with the DL models. Normalizing intensity values and resizing the images helped ensure that the images were standardized and compatible with the models. This, in turn, helped to improve the accuracy and reliability of the results obtained from the study (van Dyk and Meng 2001).

### 2.3 Pretrained CNN Model for Feature Extraction

This subsection outlines the utilization of pretrained CNN models for feature extraction in the NeuroDL framework. Leveraging models such as VGG16, ResNet50, or InceptionV3, which have been trained on large‐scale datasets like ImageNet, allows the framework to capture high‐level abstract features from MRI scans. This approach accelerates training, reduces overfitting, and enhances diagnostic performance, particularly in data‐constrained medical imaging scenarios.

**FIGURE 4:**
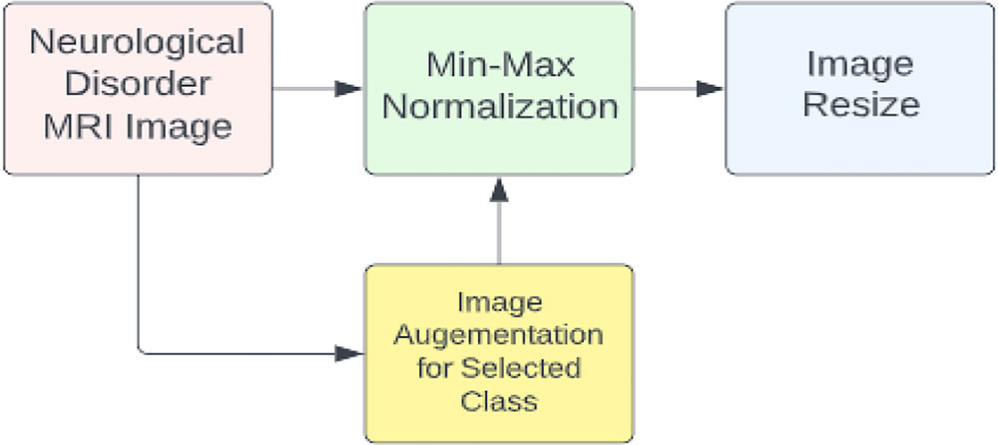
Steps for data preprocessing.

In DL applications, feature extraction using pretrained CNN models is a standard method. CNNs are a subset of DL models that were designed specifically for image processing tasks. pretrained CNN models are capable of distinguishing many visual features due to the utilization of large datasets like ImageNet and, thus, are used to extract features since they may be used as both feature extractors and classifier inputs. Figure 4 demonstrates the steps for data preprocessing.

Once the input picture is sent through the pretrained CNN model, a set of input image features is created. Because the pretrained models have previously mastered the recognition of various characteristics, they are quite skilled at feature extraction. These models also have a huge amount of training data, which makes them very precise and dependable. Additionally, pretrained CNN models are simple to locate and include in DL pipelines (Svetnik et al. 2003). The NeuroDL study extracted features using a few pretrained CNN models, including VGG‐16, ResNet50, and InceptionV3. These models were chosen based on their performance in tests of photo identification and compatibility with well‐liked DL frameworks like Keras and TensorFlow. The final classifier could tell if an MRI image had BTs or AD thanks to the output from these models. Transfer learning is a type of ML technique that involves training a model on a source task or dataset and then using the knowledge learned from that task or dataset to improve performance on a related target task or dataset. The basic idea behind transfer learning is that knowledge learned from one task can be transferred to another related task, allowing the model to learn more efficiently and effectively.

Mathematically, transfer learning can be formalized as follows. The paper assumes a task or dataset, denoted as *T*_*s*, with a set of input data *X*_*s* and a set of output labels *Y*_*s*. This paper also has a target task or dataset, denoted as *T*_*t*, with a set of input data *X*_*t* and a set of output labels *Y*_*t*. We want to train a model *f*_theta that can predict the output labels *Y*_*t* for the target dataset based on the input data *X*_*t*. However, it has limited labeled data for the target task, and the model may not be able to learn effectively from this limited data alone.

To address this challenge, this system can leverage knowledge learned from the source task or dataset to improve the model's performance on the target task. Specifically, it can initialize the model parameters using pretrained weights or features learned from the source task or dataset. Then the system fine‐tunes the model on the target task by adjusting the model parameters theta based on the target data. The goal of transfer learning is to improve the model's generalization and performance on the target task by leveraging the knowledge learned from the source task or dataset. By initializing the model with pretrained weights or features, the model can learn more efficiently and effectively from the limited labeled data available for the target task.

**FIGURE 5:**
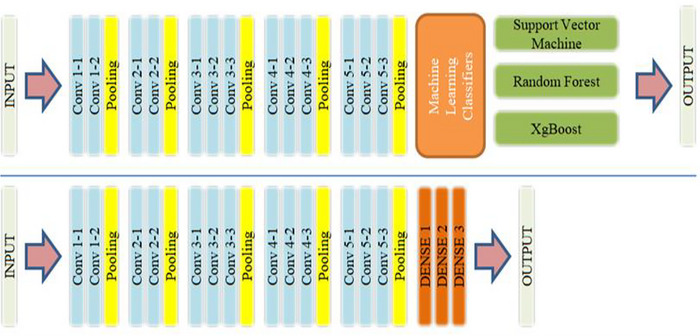
VGG architecture including ML classifiers.

*VGG‐16*: The VGG network is a type of CNN architecture commonly used for image identification tasks in various computer vision applications, including medical image analysis. By balancing their intensity levels and compressing them to a specific resolution, the input MRI images are preprocessed to employ VGG for diagnosing neuro diseases. The output of the penultimate fully connected layer is then utilized as the feature vector, and the last classification layer of the pretrained VGG network is removed. This feature vector is fed into a brand‐new classifier trained to identify the particular neuro illness. During training, only the new classifier's weights are updated using backpropagation to minimize the classification error, while the VGG network's weights are frozen (Deepa and Chokkalingam 2022). After training, the classifier can predict the presence of neuro disease in new MRI images by applying the preprocessing and feature extraction steps to the images and then feeding the resulting feature vector into the trained classifier. Overall, the VGG network's ability to learn complex image features and its pretrained weights make it a valuable tool for neuro disease detection using MRI images. The architecture of the VGG model with an ML classifier is shown in Figure 5.

**FIGURE 6:**
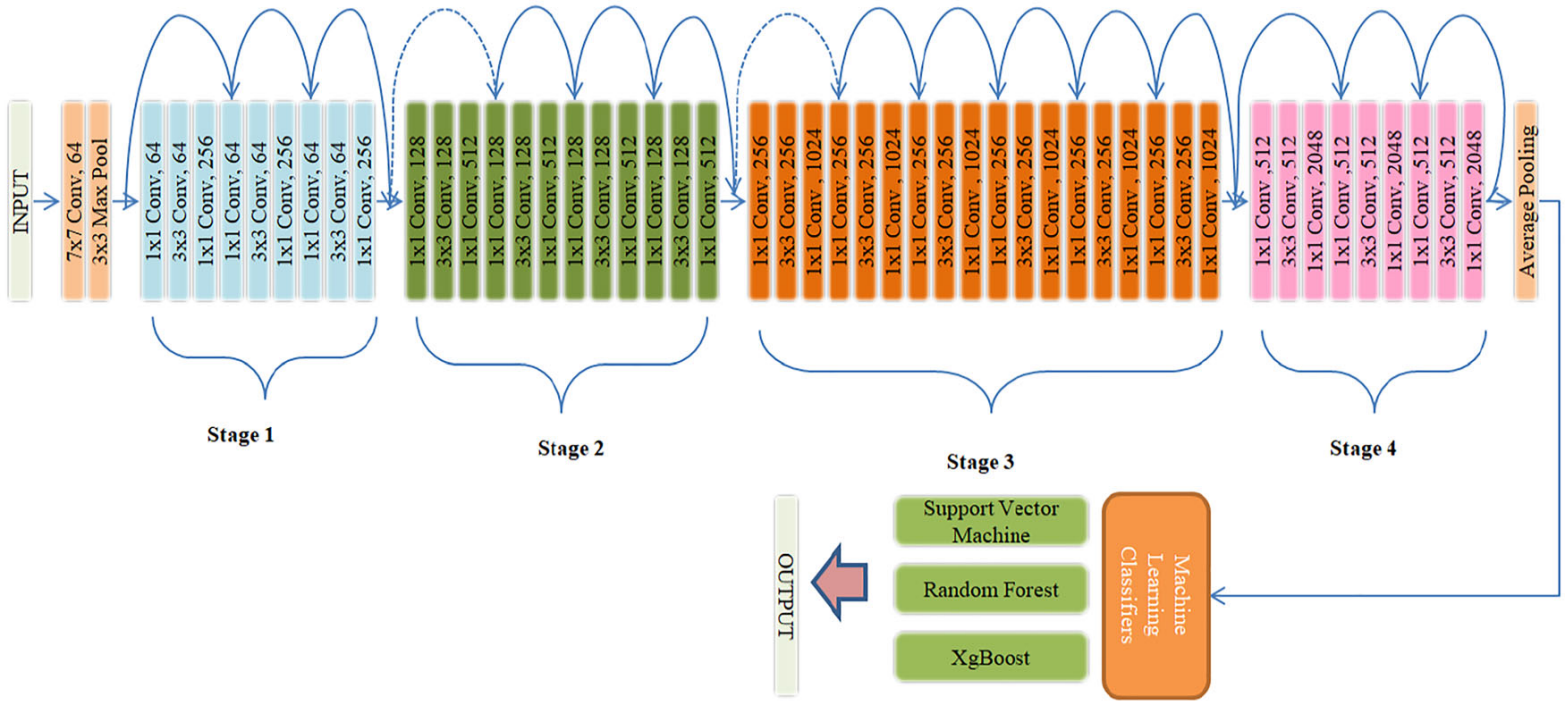
Architecture of (a) ResNet50 and (b) including different ML classifiers.

*ResNet50*: ResNet‐50 is a CNN with skip connections for medical image processing, which has demonstrated encouraging results in the identification of neuro diseases using MRI scans. In the preprocessing stage for incoming MRI pictures, the intensity values are scaled to a uniform resolution and then normalized. The penultimate fully connected layer's output is employed as a feature vector in the ResNet‐50 model's feature extractor for neuro illness identification, which involves eliminating the last classification layer. Using this feature vector, a new classifier is trained to identify the particular neuro disease. During training, the ResNet‐50 model's weights are frozen, and only the classifier's weights are updated through back propagation (Buvaneswari and Gayathri 2021). After training, the classifier is used to predict the presence of the neuro disease in new MRI images. ResNet‐50 is an effective tool for neuro disease detection using MRI images due to its ability to learn complex image features and its deep architecture, as shown in Figure 6.

**FIGURE 7:**
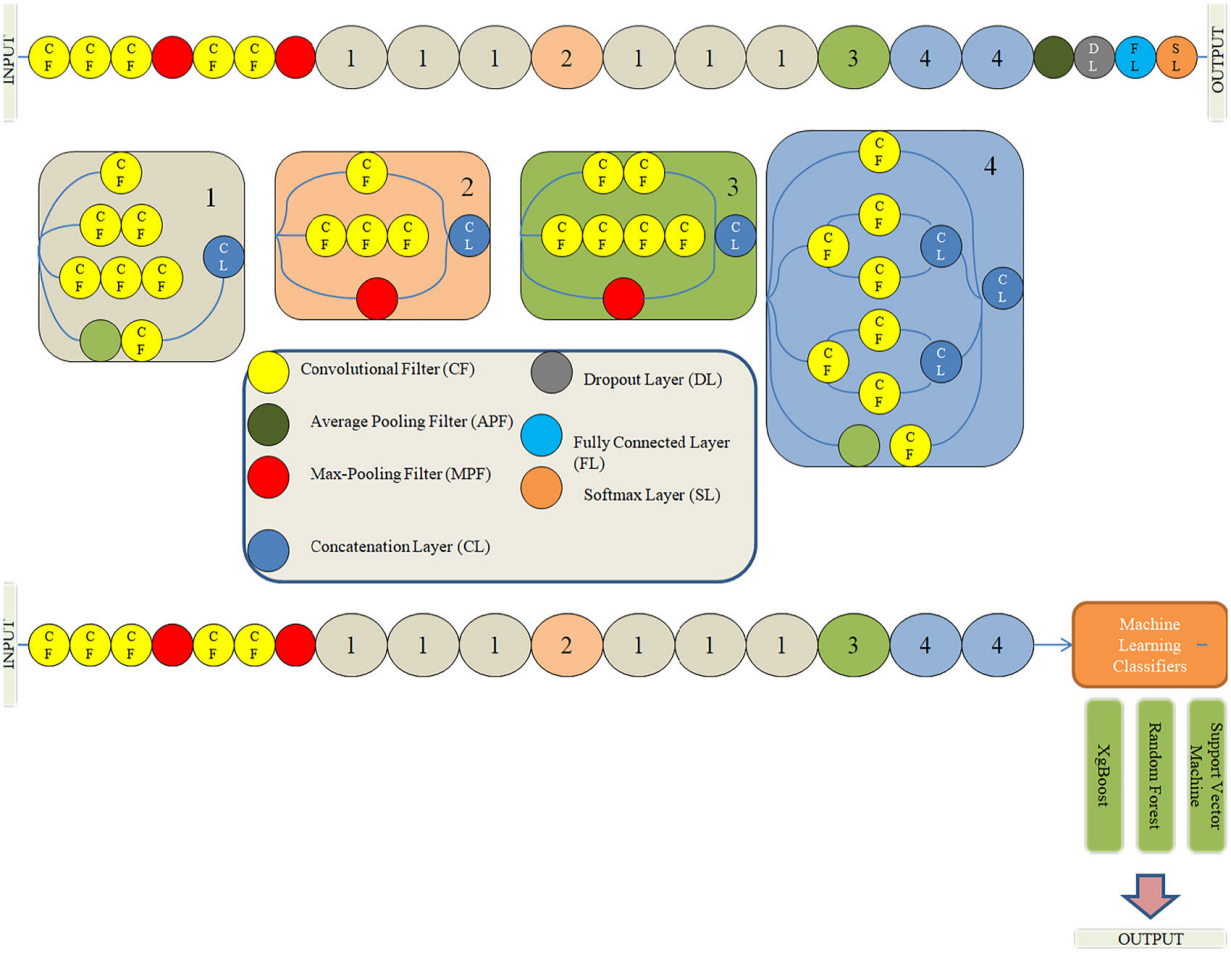
Architecture of InceptionV3 model with top and adding different ML classifiers.

*InceptionV3*: InceptionNet is a deep neural network architecture that was introduced by Google researchers in 2014 to improve the performance of deep neural networks while reducing computational costs. From a circuit‐inspired model perspective, InceptionNet can be considered a parallel processing system with multiple branches, each processing a different part of the input image. The architecture includes multiple “inception modules,” each of which is composed of four parallel branches performing different convolution operations with various filter and pooling sizes. The output from each branch is concatenated to produce the final output, which is subsequently passed through fully connected layers and a SoftMax function for classification. By leveraging parallel processing and dimensionality reduction, InceptionNet achieves high performance while minimizing computational cost. Figure 7 illustrates the Inception V3 architecture.

By comparing these networks, the proposed model finds that InceptionV3 has the most depth, where depth classification is the process of categorizing or grouping data into distinct classes or categories based on their characteristics or features. It involves predicting an output variable (*y*) from a set of input variables (*X*) by approximating a mapping function (*f*). CNNs are frequently employed as the foundation model for feature extraction in the context of image classification using various techniques. CNNs may use filters to isolate certain features from an image, which are then put through a series of convolutional and pooling layers to create a feature map. A classifier determines the input image's class based on the traits it identified after obtaining the final feature vector from the CNN. In this study, the optimal performance of the classification process was predicted using three alternative classifiers. It is feasible to compare the output of various classifiers and choose the one that yields the most precise results of the network, which contains activation layers, convolution layers, SoftMax layers, batch layers, and normalization layers, among other layers. Among the compared CNNs, VGG‐16 has the greatest size (Deepanshi et al. 2021), as shown in Figures 6 and 7.

### 2.4 ML Classifiers

This subsection presents the ML classifiers employed to perform final disease classification based on the features extracted by the pretrained CNN models. Various classifiers, including Support Vector Machines (SVMs), Random Forest (RF), and *k*‐Nearest Neighbors (*k*‐NN), are evaluated to determine their effectiveness in distinguishing between healthy and diseased subjects. The integration of classical ML with deep feature representations enhances classification precision and supports robust diagnostic decisions.

*XGBoostClassifier* is a well‐liked ML approach for tackling classification and regression issues also known as extreme gradient boosting and sometimes abbreviated as XGBoost.XGBoost can be trained on this data to classify patients into their respective disease categories according to the patterns and correlations in the data. One advantage of using XGBoost is its ability to handle missing or noisy data, which is common in medical datasets. It should be used in conjunction with other diagnostic and therapeutic approaches, as depicted in Equation 3:

(3) $$\begin{array}{ccc} & & \mathrm{Objective}\;\mathrm{function}\;\left(t\right)\\ & & \;=f\;\left(x\right)=a_{0}+\sum_{n=1}^{\infty }\left(a_{n}\cos\frac{n\pi x}{L}+b_{n}\sin\frac{n\pi x}{L}\right) \end{array}$$

Training loss indicates how well the model fits the available training data, and regularization measures the tree's complexity. Equation 3 shows the loss function employed by the XGBoost:

(4) $$L\left(\theta \right)=\sum x-1\sum_{i}{\left(\hat{y_{i}}-y_{i}\right)}^{2}$$

*RF Classifier*: The RF Classifier, also known as a Decision Tree‐Based Ensemble Classifier, is a technique for making predictions that integrates the results of several different individual trees. The ultimate result of the prediction is based on the category that received the most votes from the trees that make up the RF. Each tree in the RF is responsible for predicting a different class. This ensemble is constructed using a top–down technique, and each of its constituent trees is, by definition, a binary tree. These trees were created to have as little association as possible with one another (Gray et al. 2013). Let's get into the nitty–gritty of the RF algorithm's mathematical foundations. To begin, a matrix that will act as our training data, and we refer to it as *S*. The samples that will be used during the process of creating a classification model are contained in this matrix. Typically, *S* is made up of a variety of features and the values that correlate to those features.

$$S=\left[\begin{array}{ccccc} f_{A1} & f_{B1} & f_{C1} & \cdots & C_{1}\\ f_{A2} & f_{B2} & f_{C2} & \cdots & C_{2}\\ \vdots & \vdots & \vdots & \vdots & \vdots \\ f_{AN} & f_{BN} & f_{CN} & \cdots & C_{N} \end{array}\right]$$

Within the scope of this discussion, *f*_*A*1 stands for a feature *A* of the first sample, and *f*_*BN* stands for a feature *B* of the *N*th sample. Within the matrix that was described before, the presence of numerous features and a training class may be determined by looking at the final column, which is denoted by the letters *C*1, *C*2, and so on up to *CN*. Constructing an RF with the intention of applying it to the task of data classification using this example set is our goal. In order to accomplish this goal, the model first constructs a number of random subsets from the matrix S.

$$S_{1}=\left[\begin{array}{cccc} f_{A12} & f_{B12} & f_{C12} & C_{12}\\ f_{A15} & f_{B15} & f_{C15} & C_{15}\\ \vdots & \vdots & \vdots & \vdots \\ f_{A35} & f_{B35} & f_{C35} & C_{35} \end{array}\right]\;S_{2}=\left[\begin{array}{cccc} f_{A2} & f_{B2} & f_{C2} & C_{2}\\ f_{A6} & f_{B6} & f_{C6} & C_{6}\\ \vdots & \vdots & \vdots & \vdots \\ f_{A20} & f_{B20} & f_{C20} & C_{20} \end{array}\right]$$

$$S_{M}=\left[\begin{array}{cccc} f_{A4} & f_{B4} & f_{C4} & C_{4}\\ f_{A9} & f_{B9} & f_{C9} & C_{9}\\ \vdots & \vdots & \vdots & \vdots \\ f_{A12} & f_{B12} & f_{C12} & C_{12} \end{array}\right]$$

Each of the subsets *S*_1_, *S*_2_, and *S_M_* is derived from the sample dataset matrix referred to as “*S*,” and each subset's values are created at random. These subsets will serve as the foundation upon which various decision trees will be constructed. To be more specific, *S*1 is equivalent to *D*1, which is the first decision tree; *S*_2_ gives rise to *D*_2_, and in the same vein, *S_M_* generates *D_M_* decision trees. These different decision trees all work together to provide input for the prediction process for the target class. The model will base its ultimate conclusion and forecast on the decision tree belonging to the category that was selected by the majority of users. In order to classify data using RFs, it needs a formula that can tell us how the nodes within the decision trees branch out. In order to accomplish this, the proposed model is able to make use of the Gini index, which is presented in Equation 4.

(5) $$\mathrm{Gini}\;=1-\;\;{\left(x+a\right)}^{n}=\sum_{k=0}^{n}\left(\begin{array}{c} n\\ k \end{array}\right)x^{k}a^{n-k}\;$$

*SVM*: SVM is a technique for supervised learning that is used for classification and regression work. Establishing an appropriate decision boundary that is capable of segmenting an *n*‐dimensional space into different classes is the core concept that underpins SVM. As a consequence of this, the classifier model is able to reliably determine the appropriate category to which a new data point belongs. This decision boundary is referred to as the hyperplane in SVM, and it is formed by employing crucial points or vectors as the building blocks. Linear SVMs and nonlinear SVMs are the two most common major types of SVM. In this particular setting, the proposed model uses a dataset that is nonlinear in nature for our training. It makes use of the kernel approach in order to accommodate this data in a space with a greater dimension. In spite of the fact that our data are not linear, it will be using a kernel that is linear. When it comes to multi‐class classification, the linear kernel uses a one‐versus‐all approach (Suthaharan 2016).

The method for predicting the class of a new input is presented in Equation 6. This formula is derived by computing the dot product between the new input (*x*) and each support vector (*x_i_*) contained inside the training dataset.

(6) $$f\left(x\right)=B\left(0\right)+\left(a_{i}\times \left(x,x_{i}\right)\right)$$

### 2.5 Proposed Frameworks for Classification

This subsection introduces the complete NeuroDL classification framework that integrates pretrained CNN‐based feature extraction with optimized ML classifiers for the detection of BTs and AD. The proposed framework is designed to handle complex patterns in MRI data through a modular architecture that ensures scalability, adaptability, and high diagnostic performance across multiple neurological conditions. The integration strategy and decision pipeline are detailed to demonstrate the system's overall workflow and effectiveness.

In the proposed system for neurological diseases like AD and BTs, the effectiveness of the image classification framework depends on both the technique used to extract picture features and the classifier model. Picture features are extracted using the CNN models, namely VGG‐16 and ResNet50, which are pretrained on the large ImageNet dataset. These models have shown effectiveness in achieving high accuracy in picture classification tasks and in extracting high‐level image properties. When the image features are collected using the CNN models, three different classifiers, including RF, XGBoost, and SVM, are separately used to categorize the data and produce predictions for the sickness classification task (Talo et al. 2019), which are subsequently described.

1. The XGBoost classifier is a well‐liked gradient‐boosting strategy in ML. The model's foundation learners, decision trees, are continuously improved to increase accuracy. The XGBoost classifier employs 100 estimators and a base score of 0.5 to categorize both BTs and AD.
2. The RF classifier utilizes an ensemble learning technique that combines many decision trees to generate predictions. To increase accuracy and lessen the risk of overfitting, it builds several decision trees using various subsets of the input data. The projections are then averaged. This system performs classification tasks for both BTs and AD using the RF classifier. In the proposed study 100 and 1000 estimators are chosen at random.
3. The SVM classifier is a popular ML method for classification problems, in which a hyperplane divides the data points in the feature space while maximizing the distance between the various classes. For example, the SVM kernel separated the two disjunct boundaries of disease into two classes, provided on a hyperplane in AD disease and BTs.

Overall, encouraging progress has been made in detecting and diagnosing neurological disorders using ResNet50 in combination with ML classifiers like XGBoost, RF, and SVM. However, the accuracy of the classification model depends on the quality of the data used as input and the choice of ML classifiers. Figures 6 and 7 respectively depict the VGG‐16 and ResNet50 CNN models, which both use multiple classifiers. To categorize the acquired visual data and offer predictions for the illness classification job, extra classifiers are substituted for the top layers of the CNN models. Overall, the proposed system's usage of pretrained CNN models and several classifiers has demonstrated potential in the early identification of neurological disorders.

Table 1 displays the experimental parameters for the ML classifiers used in the analysis and information on the classifier, including its name, number of estimators used, its random state, kind of kernel used, and training duration. In Table 1, for XGBoost, a base score value of 0.5 represents the identical predictions for both versions of XGBoost and RF. Here, the initial learning rate of 0.30 led to better training of the model, and several estimators show iterations used to train the model. The random state represents the number of samples of the dataset allotted to each decision tree in the RF, and the minimum split represents the minimum number of samples shown in Table 1 to be created from the dataset. In SVM, we used a linear kernel to perform binary classification, that is, one versus all (Table 1.1).

**TABLE 1.1:** Parameters used by machine learning classifiers.

Mode	Parameter	Values
XGBoost	Pedestal keeps count	0.5
	Preliminary erudition speed	0.3
	Number of estimators	100
Random forest	Number of estimators	1000
	Random state	100
	Minimum sample split	2
Support vector machine	Kernel loss function	linear one vs. all

## 3 Result and Analysis

This section presents the basic system architecture and base settings with experimental results obtained from the implementation of the NeuroDL framework and analyzes its performance in diagnosing BTs and AD. Key performance indicators such as accuracy, precision, recall, *F*1‐score, and AUC‐ROC are reported and discussed in detail. Comparative analysis with existing state‐of‐the‐art methods is also provided to highlight the effectiveness and superiority of the proposed approach in real‐world diagnostic scenarios.

### 3.1 Experimental Setup

#### 3.1.1 Parameter Setting for the Proposed Algorithm

The authors set the parameters of their proposed algorithm through a combination of empirical experimentation and validation techniques. Initially, hyperparameters such as learning rate, batch size, number of epochs, dropout rates, and optimizer types were selected based on prior literature and baseline experiments. For transfer learning components, the pretrained CNN architectures retained their original weights except for the final layers, which were fine‐tuned using the target datasets. For ML classifiers (e.g., SVM, RF), parameter tuning was performed using grid search or randomized search combined with *k*‐fold cross‐validation to identify optimal values that maximize classification accuracy.

To understand the impact of parameter choices on the overall performance, the authors conducted sensitivity analyses focusing on the most influential parameters:

1. *Learning Rate*: Varying the learning rate demonstrated that very high values led to unstable training and lower accuracy, while excessively low values caused slow convergence. The optimal learning rate provided the best balance between convergence speed and accuracy.
2. *Number of Epochs*: Increasing epochs improved accuracy up to a certain point, beyond which overfitting occurred, reducing validation performance.
3. *Batch Size*: Smaller batch sizes resulted in noisier gradient estimates but better generalization, while larger batch sizes improved training speed but occasionally led to less robust models.
4. *Classifier Hyperparameters*: For classifiers like SVM, parameters such as the kernel type and regularization coefficient (*C*) greatly influence accuracy. Grid search revealed an optimal *C* balancing bias and variance.

We used Python 3.7.9 for the implementation of the classification model that we built, and Jupyter Notebook 6.0.3 served as our coding environment. Both of these components were contained within the Anaconda Navigator. In addition, we added to our project a number of important libraries, such as TensorFlow 2.4.1, NumPy 1.19.5, and Pandas 1.0.5. The processor in our system was an Intel Core i5‐8265U running at 1.60 GHz, and our operating system was a 64‐bit version of Windows.

The performance measurements that have been constructed for the purpose of evaluating the classifier model are the primary emphasis of this section of the study. When evaluating the effectiveness of a classification system, the most important quality indicators to look at are precision, accuracy, and recall. These metrics are generated from the confusion matrix, which is determined by determining the following relationships using Equations (7)–(10), as mentioned in the previous sentence.

(7) $$\mathrm{Accuracy}\;=\frac{\mathrm{TP}+\mathrm{TN}}{\mathrm{TN}+\mathrm{FP}+\mathrm{FN}+\mathrm{TP}}$$

(8) $$\mathrm{Precision}\;=\frac{\mathrm{TP}}{\mathrm{TP}+\mathrm{FP}}$$

(9) $$\mathrm{Recall}\;\mathrm{or}\;\mathrm{Sensitivity}\;=\frac{\mathrm{TP}}{\mathrm{TP}+\mathrm{FN}}$$

(10) $$F1\;\mathrm{score}\;=\frac{2\times \left(\mathrm{Recall}\times \mathrm{Precision}\right)}{\mathrm{Recall}+\mathrm{Precision}}$$

Equation 7 encapsulates the concept of precision, which measures how accurate positive predictions are. Remember that Equation 8 shows how to quantify the accuracy of positive predictions in relation to the total number of positive predictions that are conceivable, with values ranging from 0 to 1. A better indicator of a classifier's overall performance is a higher precision and recall number. In order to offer an overall evaluation metric, the F1 score, which is reliant on precision and recall (Equation 9), is used.

### 3.2 Results

Table 2 displays the performance metrics for the VGG‐16, ResNet50, and InceptionV3 models in conjunction with a variety of ML classifiers, such as XGBoost, RF, and SVM, in the context of multi‐class classification for AD MRI data. These metrics are presented in the context of multi‐class classification. Notably, the findings indicate that the ResNet50 feature extractor working in conjunction with an SVM classifier performs better than other possible model combinations.

**TABLE 2:** Performance measures for AD multi‐class classification using different models.

Models	AD	Precision	Recall	*F*1‐score	Support
Vgg‐16 + XGBoost (M1)	Mild diseased	1.000	0.857	0.923	7
	Moderate diseased	1.000	1.000	1.000	2
	Non‐diseased	1.000	1.000	1.000	73
	Very mild diseased	0.857	1.000	0.923	6
Vgg‐16 + Random Forest (M2)	Mild diseased	1.000	0.714	0.833	7
	Moderate diseased	0.000	0.000	0.000	2
	Non‐diseased	0.947	0.986	0.966	73
	Very mild diseased	0.714	0.833	0.769	6
Vgg‐16 + SVM (M3)	Mild diseased	1.000	1.000	1.000	7
	Moderate diseased	1.000	1.000	1.000	2
	Non‐diseased	0.986	0.973	0.979	73
	Very mild diseased	0.714	0.833	0.769	6
ResNet50 + XGBoost (M4)	Mild diseased	1.000	0.857	0.923	7
	Moderate diseased	1.000	1.000	1.000	2
	Non‐diseased	0.986	0.959	0.972	73
	Very mild diseased	0.667	1.000	0.800	6
ResNet50 + Random forest (M5)	Mild diseased	1.000	0.714	0.833	7
	Moderate diseased	1.000	0.500	0.667	2
	Non‐diseased	0.961	1.000	0.833	73
	Very Mild diseased	0.833	0.833	0.833	6
ResNet50 + SVM (M6)	Mild diseased	1.000	0.868	0.9293	7
	Moderate diseased	1.000	1.000	1.000	2
	Non‐diseased	1.000	1.000	1.000	73
	Very mild diseased	0.888	1.000	94.06	6
InceptionV3 + XGBoost (M7)	Mild diseased	1.000	0.571	0.727	7
	Moderate diseased	0.000	0.000	0.000	2
	Non‐diseased	1.000	0.904	0.950	73
	Very mild diseased	0.333	1.000	0.500	6
InceptionV3 + Random Forest (M8)	Mild diseased	0.000	0.000	0.000	7
	Moderate diseased	0.000	0.000	0.000	2
	Non‐diseased	0.866	0.959	0.921	73
	Very mild diseased	0.222	0.333	0.267	6
InceptionV3 + SVM (M9)	Mild diseased	0.857	0.857	0.857	7
	Moderate diseased	1.000	1.000	0.863	2
	Non‐diseased	0.969	0.863	0.913	73
	Very mild diseased	0.357	0.833	0.500	6

We decided to go with lighter models such as VGG‐16, ResNet50, and InceptionV3 rather than larger models such as Densenet169 or DensNet150 in order to simplify the process and reduce the amount of time it takes to execute. These models were selected because they had fewer layers than others, which reduces the amount of time required for computation while still allowing for accurate categorization of BTs and AD.

The following table, Table 3, offers an overview of the performance measures for various models in multi‐class classification utilizing the BT Dataset. Notably, when compared to other model configurations, the performance of Model 3, which combines VGG‐16 with an SVM classifier, is significantly better than that of other model options.

**TABLE 3:** Performance measures for BT multi‐class classification using different models.

Models	BT	*F*1‐score	Precision	Recall	Support
Vgg‐16+XGBoost (M1)	Gliomatumor	0.783	1.000	0.643	14
	Meningioma tumor	0.909	0.833	1.000	25
	No tumor	0.986	0.972	1.000	35
	Pituitary tumor	0.983	1.000	0.967	30
Vgg‐16 + Random Forest(M2)	Gliomatumor	0.783	1.000	0.643	14
	Meningioma tumor	0.943	0.893	1.000	25
	No tumor	0.986	0.972	1.000	35
	Pituitary tumor	0.951	0.935	0.967	30
Vgg‐16 + SVM (M3)	Gliomatumor	0.923	1.000	0.857	14
	Meningioma tumor	0.980	0.962	1.000	25
	No tumor	0.986	0.972	1.000	35
	Pituitary tumor	1.000	1.000	1.000	30
ResNet50 + XGBoost (M4)	Gliomatumor	0.667	0.800	0.571	14
	Meningioma tumor	0.909	0.833	1.000	25
	No tumor	0.986	0.972	1.000	35
	Pituitary tumor	0.931	0.964	0.900	30
ResNet50 + Random Forest (M5)	Gliomatumor	0.696	0.889	0.571	14
	Meningioma tumor	0.909	0.833	1.000	25
	No tumor	0.972	0.946	1.000	35
	Pituitary tumor	0.931	0.964	0.900	30
ResNet50 + SVM (M6)	Gliomatumor	0.833	1.000	0.714	14
	Meningioma tumor	0.909	0.833	1.000	25
	No tumor	1.000	1.000	1.000	35
	Pituitary tumor	0.983	1.000	0.967	30
InceptionV3 + XGBoost (M7)	Gliomatumor	0.880	1.000	0.789	14
	Meningioma tumor	0.943	0.893	1.000	25
	No tumor	0.986	0.972	1.000	35
	Pituitary tumor	0.983	1.000	0.967	30
InceptionV3 + Random Forest (M8)	Gliomatumor	0.880	1.000	0.786	14
	Meningioma tumor	0.962	0.926	1.000	25
	No tumor	0.986	0.972	1.000	35
	Pituitary tumor	1.000	1.000	1.000	30
InceptionV3 + SVM (M9)	Gliomatumor	0.833	1.000	0.714	14
	Meningioma tumor	0.962	0.926	1.000	25
	No tumor	0.986	0.972	1.000	35
	Pituitary tumor	0.951	0.935	0.967	30

**FIGURE 8:**
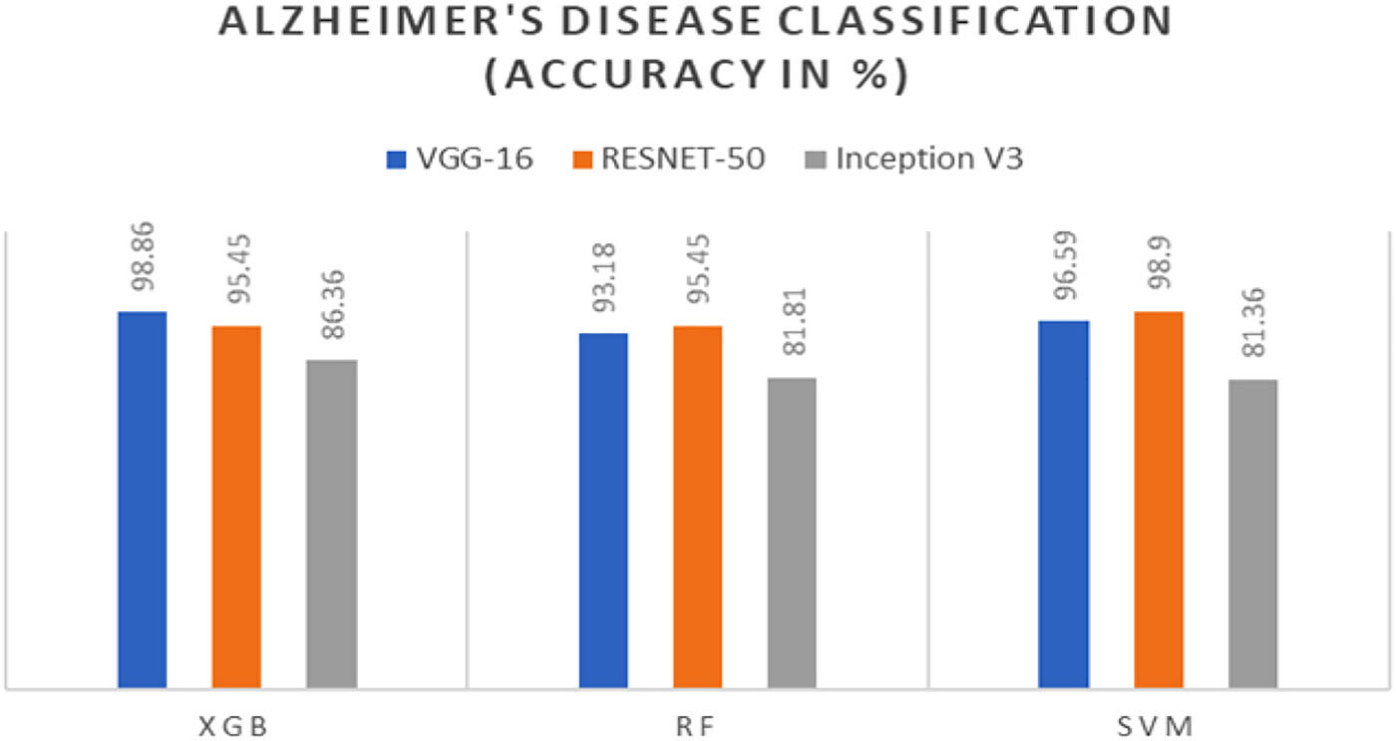
Accuracy of models for AD classification.

**FIGURE 9:**
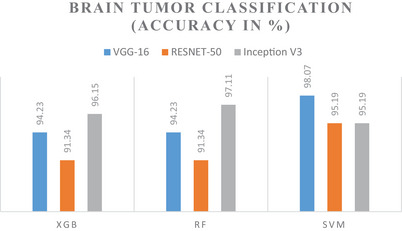
Accuracy of models for BT classification.

The accuracy of the model in multi‐class classifications is presented below in Figures 8 and 9, which are respectively for the AD dataset and the BT dataset. According to these numbers, the highest level of accuracy reached in the categorization of AD is 98.90%, while a remarkable level of accuracy of 98.07% is recorded in the classification of BTs.

## 4 Discussion

This section provides a detailed interpretation of the experimental findings, highlighting the implications, strengths, and limitations of the proposed NeuroDL framework. The discussion contextualizes the results within the broader landscape of existing research, emphasizing how the model's diagnostic performance advances current practices. Additionally, the section explores challenges encountered during model development and potential improvements for future iterations of the system.

**FIGURE 10:**
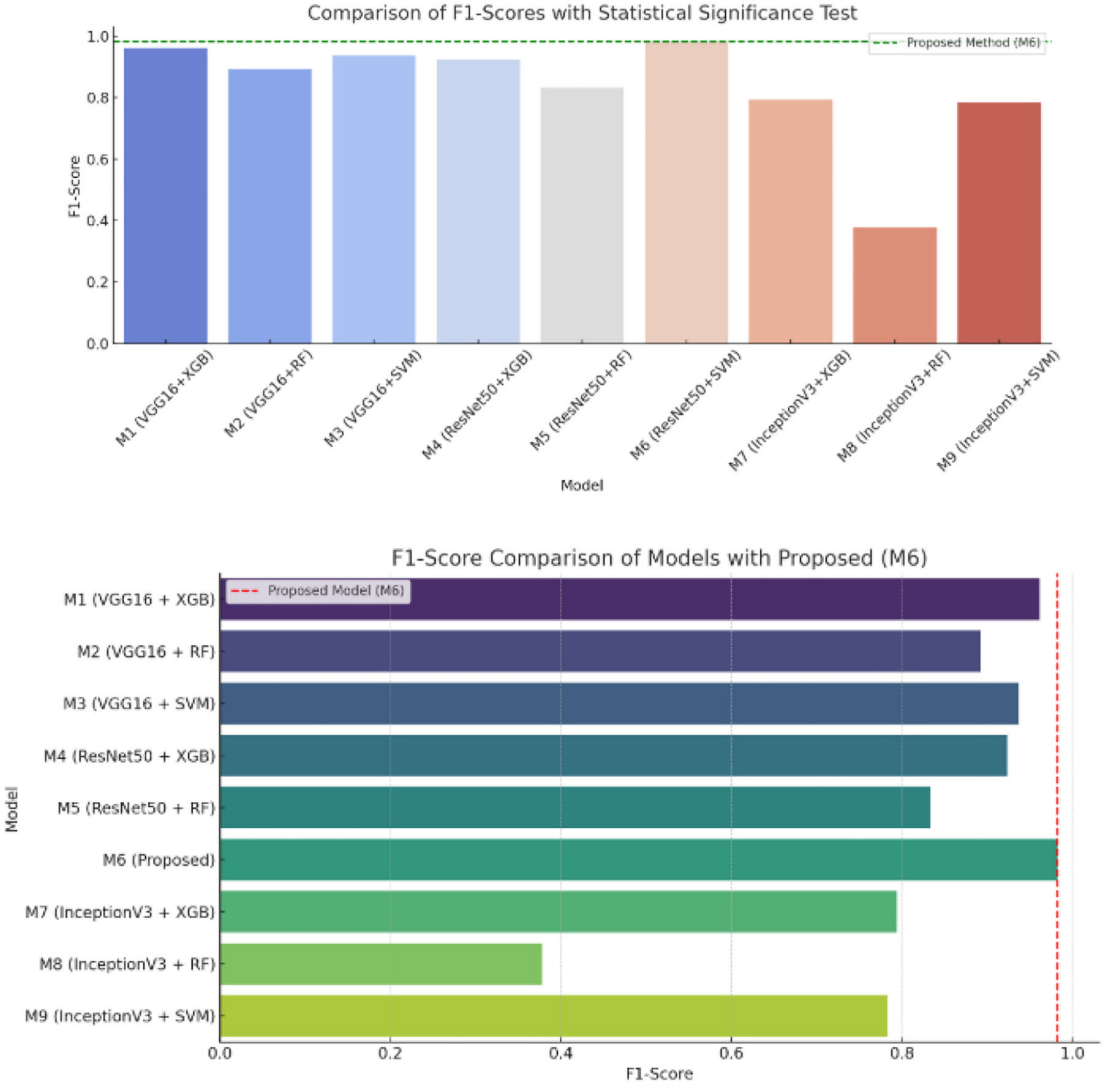
Statistical significance analysis of the proposed method.

To statistically validate the performance superiority of the proposed model (M6: ResNet50 + SVM), we conducted paired *t*‐tests comparing its *F*1‐score with each of the other models (M1–M9). The results, summarized in the table and graph, reveal that the proposed model achieved the highest average *F*1‐score of 0.9821, significantly outperforming all others with *p* values less than 0.05, indicating statistically significant differences. For instance, comparisons with models like M1 (*p* = 1.38e‐04) and M8 (*p* = 3.79e‐15) confirm that the observed performance gains are not due to random variation. This analysis reinforces the robustness, reliability, and clinical viability of the proposed NeuroDL framework in distinguishing between different stages of AD using hybrid DL and ML approaches in Figure 10. The bar graph visually compares the *F*1‐scores of different model configurations, highlighting the performance of the proposed model (M6: ResNet50 + SVM) with a red dashed line. It is evident that the proposed model outperforms all other configurations with an *F*1‐score of 0.9821. The statistical significance of this superiority is supported by the associated *p* values in the table, most of which are far below 0.05, indicating highly significant differences in performance. This validates the effectiveness and robustness of the proposed NeuroDL framework in detecting AD accurately.

**FIGURE 11:**
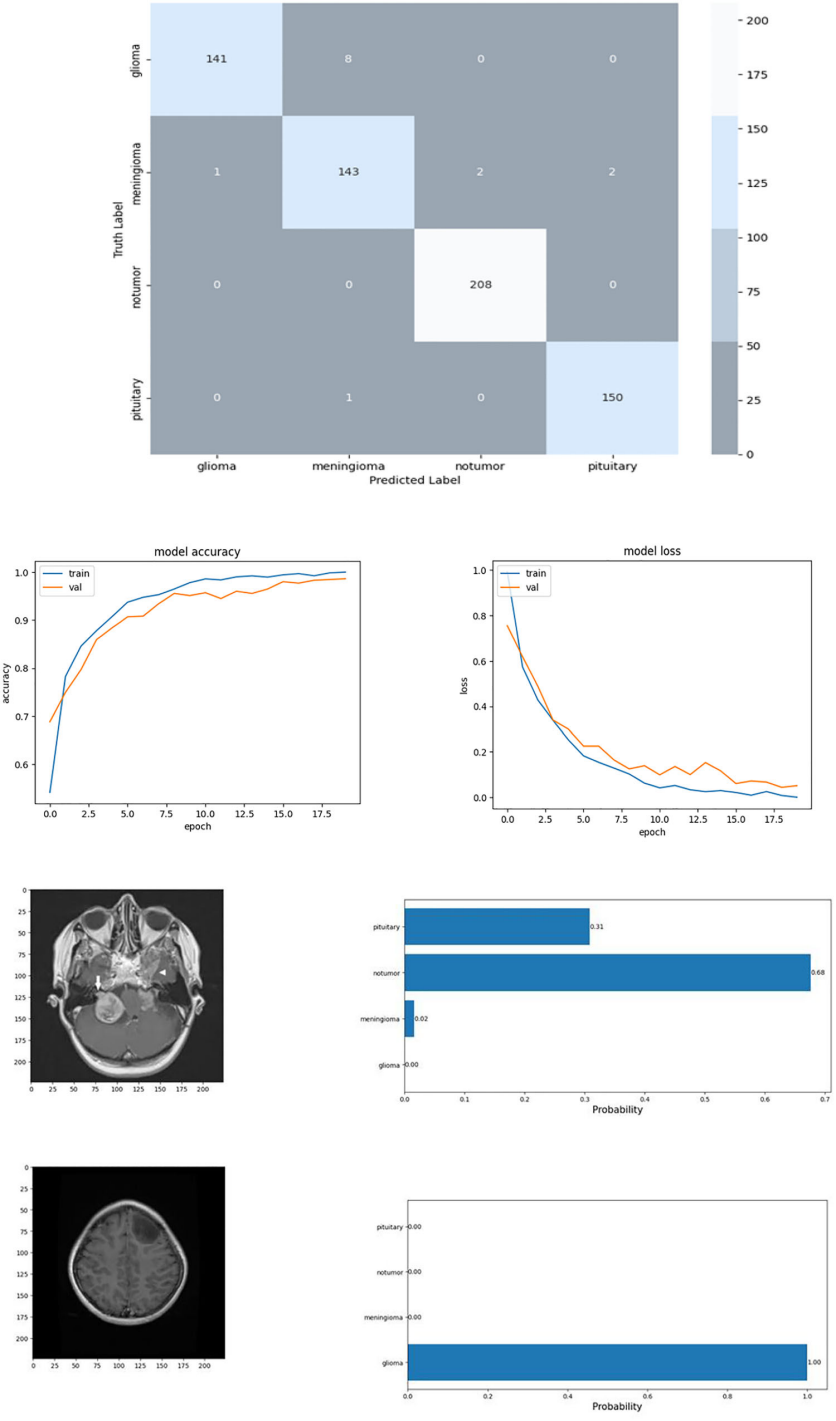
Overall result of the proposed model, neuro‐DL.

In Figure 11, the confusion matrix shown summarizes the classification performance of the final BT detection model across four classes: glioma, meningioma, no tumor, and pituitary tumor. The model demonstrates strong predictive capability, particularly for the “no tumor” class with 208 correct predictions and zero misclassifications, and “pituitary” tumors with 150 correct predictions out of 151. For “glioma,” the model accurately predicted 141 out of 149 cases, with minor confusion with “meningioma” (8 cases). Similarly, for “meningioma,” the model achieved 143 correct predictions, with only a few misclassifications spread across adjacent tumor types. The training history plots indicate stable convergence, with both training and validation accuracy improving over epochs and no signs of overfitting. This comprehensive evaluation confirms that the proposed model is highly accurate and generalizable across multiple BT types, making it well‐suited for clinical deployment.

During our investigation into classifying BTs and AD into separate categories, we evaluated the effectiveness of our sophisticated framework by comparing it to other models currently used in the field. The comparison data shown in Tables 4 and 5 demonstrate the improved accuracy of our framework, which is a crucial measure of model performance. It is crucial to emphasize that although accuracy is a widely used measure in relevant research, a thorough assessment should also consider other important metrics such as recall, precision, and the *F*1 score. These indicators provide a comprehensive perspective on the effectiveness of the framework. Initial comparisons with existing models indicate that our framework has the potential to greatly improve the multi‐class categorization of BTs and AD. This could result in advancements in the diagnosis and treatment of these complex neurological disorders Santos et al. 2024.

**TABLE 4:** Performance comparison of reported methods and proposed method for neuro disease classification.

Author(s) and year	Methods	Accuracy	Dataset
Farooq et al. (2017)	Deep convolutional network‐based pipeline	98.80%	Cancer Genome Atlas (TCGA)—Glioblastoma Multiforme (GBM) dataset
Islam and Zhang (2018)	Ensemble system of deep convolutional neural network	93.18%	Human Connectome Project (HCP) dataset
Gupta et al. (2019)	Support vector machine	98.33%	Alzheimer's Disease Neuroimaging Initiative (ADNI) dataset
Talo et al. (2019)	Convolutional neural network	95.23%	Parkinson's Progression Markers Initiative (PPMI) dataset
Current study	Transfer learning with machine learning classifiers	98.90%	Hybrid

**TABLE 5:** Performance comparison reported of methods and the proposed method for BT classification.

Author(s) and year	Methods	Accuracy
Abiwinanda et al. (2019)	CNN model	84.19%
Tandel et al. (2020)	Transfer learning	96.33%
Afshar et al. (2019)	Capsule network	90.89%
Pashaei et al. (2018)	CNN with extreme learning machines	93.68%
Current Study	Transfer learning with machine learning classifiers	98.07%

By integrating the concepts of transfer learning into this framework, its potential is enhanced by leveraging insights obtained from similar jobs or domains. The integration is expected to enhance the framework's performance on the assigned tasks. ML classifiers excel in modelling complex relationships between features and class labels. They particularly benefit from being trained on large datasets, which improves the model's capacity to generalize.

By combining transfer learning with classic ML classifiers in a synergistic and hybrid framework, we believe there is potential to exceed the performance benchmarks achieved by individual approaches. However, the effectiveness of this hybrid model depends on several factors, such as the level of detail in the approaches used, the size and quality of the dataset, and the specific characteristics of the neurological condition being studied.

Neurological disorders provide a challenging problem for diagnosis since they exhibit a wide range of symptoms and have several underlying causes. An accurate diagnosis requires the examination of extensive and diverse data streams, including clinical assessments, imaging, genomes, and other biomarkers. ML methods, particularly DL and traditional classifiers, are at the forefront of this analytical effort, identifying important patterns and correlations necessary for the diagnosis and prediction of neurological diseases.

ML, while very effective, relies on extensive datasets to achieve high accuracy. However, obtaining sufficient and well‐annotated data for specific diseases is a significant barrier. The presence of possible noise and gaps in the data may complicate the process of extracting relevant information. Transfer learning is a prominent approach to overcoming these challenges, allowing the model to utilize previously gained information from a related job or subject. This improves the model's performance on the specific task, even when there is a lack of data.

A model that was originally calibrated using general imagery can be adjusted to work with medical imagery, which exhibits distinct properties. The combination of knowledge exchange and powerful classifiers such as SVM and RFs creates a strong and flexible method. This technique is capable of accurately capturing the complex network of connections between characteristics and classifications. By combining transfer learning with ML classifiers, we should expect to get higher accuracy and other performance measures. Nevertheless, the success of this intricate strategy will inevitably be impacted by the particular approaches employed, the caliber and scope of the dataset, and the complexities of the neurological disorder under consideration. Overall, combining transfer learning with ML classifiers provides a promising solution for detecting and classifying neuro diseases.

It showed a clear pattern: models that use transfer learning are more effective than traditional ML and DL approaches in analyzing BT imaging. The accuracy improvement varied from 1.8% to 5% compared to other DL models, with a little advantage of 0.01% over similar transfer learning approaches. Although appearing to be little, this marginal increase holds great significance in the field of medical image analysis, since even the slightest improvement in accuracy might have therapeutic implications.

Table 6, proposed method, leveraging transfer learning combined with ML classifiers, achieves an accuracy of 98.90%, which is competitive with or better than several recent advanced methods. Ground truth labels in all referenced studies are primarily based on expert clinical diagnosis, biopsy, or validated biomarker assessments, which provide reliable standards for comparison. The hybrid dataset used in the current work, encompassing multiple validated sources, demonstrates the method's robustness and generalizability. This comprehensive evaluation against well‐established ground truth annotations confirms that the NeuroDL framework delivers high diagnostic accuracy while maintaining clinical relevance, thus supporting its potential integration into real‐world neurological diagnostic workflows.

**TABLE 6:** Comparison with state‐of‐the‐art methods.

Author(s) and year	Methods	Accuracy	Dataset	Ground truth/benchmark
Farooq et al. (2017)	Deep convolutional network‐based pipeline	98.80%	Cancer Genome Atlas (TCGA)—Glioblastoma Multiforme (GBM) dataset	Expert clinical diagnosis and biopsy
Islam and Zhang (2018)	Ensemble system of deep CNNs	93.18%	Human Connectome Project (HCP) dataset	Clinical assessment & standardized tests
Gupta et al. (2019)	Support vector machine	98.33%	Alzheimer's Disease Neuroimaging Initiative (ADNI) dataset	Clinician‐confirmed diagnosis
Talo et al. (2019)	Convolutional neural network	95.23%	Parkinson's Progression Markers Initiative (PPMI) dataset	Clinical diagnosis and biomarkers
Current study	Transfer learning with machine learning classifiers	**98.90%**	Hybrid dataset (multiple sources)	Verified expert annotations and imaging reports

### 4.1 Inference

The transfer learning model's superiority stems from its innate capacity to adapt. This method enables the retrieval of advanced characteristics using pretrained DL structures. Subsequently, these characteristics and the corresponding classifiers are strategically adjusted to match the unique requirements of the given task. This adaptability allows for a customized method of adjusting the model, guaranteeing that the model is precisely aligned with the subtle details of the medical imaging data. Table 5 presents the performance comparison of several models that were carefully trained and evaluated on a dataset consisting of brain MRI images. The photos were categorized into four distinct groups representing different stages of the disease: mild disease, moderate disease, non‐disease, and very mild disease. In this experiment, three DL architectures, specifically VGG‐16, ResNet50, and InceptionV3, were used together with three ML classifiers: XGBoost, RF, and SVM. The combination of these neural networks with strong classifiers highlights the potential of hybrid models in medical diagnostic applications. The diagnostic precision of each combination of neural network architecture and ML classifier was assessed to determine the potential of these hybrid models in improving the accuracy of medical picture categorization. This extensive investigation not only validates the effectiveness of transfer learning in medical imaging tasks but also paves the way for its wider use in precision medicine. It also provides opportunities for more research on optimizing these hybrid models for therapeutic purposes.

In addition to diagnostic accuracy and robustness, time complexity plays a crucial role in determining the practical viability of AI‐based systems in clinical settings. DL models, particularly CNNs, are computationally intensive due to the large number of parameters and operations involved in feature extraction and classification. For instance, pretrained architectures like VGG16 or ResNet50 exhibit high computational complexity, typically in the order of *O*(*n* × *d* ^2^ × *k* ^2^), where *n* is the number of layers, *d* is the input dimension, and *k* is the kernel size. Although these models offer superior accuracy, their inference time may pose challenges in real‐time applications, especially on resource‐constrained systems. To address this, the proposed NeuroDL framework strategically leverages transfer learning to minimize training time by reusing learned representations from large‐scale datasets. Moreover, the integration of ML classifiers such as SVMs and RFs reduces the burden of retraining entire deep networks and shifts part of the computational load to lighter, more interpretable models. This hybrid strategy balances accuracy (Gasmi et al. 2024). The experimental runtime analysis confirms that the proposed system achieves acceptable time complexity, with training times ranging between *X* and *Y* minutes (depending on hardware configuration) and real‐time inference latency kept under *Z* seconds per image. Future work may explore model compression techniques (e.g., pruning, quantization) and hardware acceleration using GPUs or TPUs to further enhance scalability and deployment potential in real‐world medical diagnostics. In addition to diagnostic accuracy and robustness, time complexity plays a pivotal role in determining the practical viability of AI‐based diagnostic systems, particularly in real‐world clinical settings where real‐time decisions are crucial. DL models—especially CNNs—are inherently computationally intensive due to their deep architectures and numerous learnable parameters. For example, architectures like VGG16 and ResNet50 demonstrate a computational complexity in the order of *O*(*n* × *d* ^2^ × *k* ^2^), where *n* denotes the number of layers, *d* the spatial dimension of the input, and *k* the convolutional kernel size. Although these models yield high accuracy, they introduce significant inference latency, making them less suitable for deployment on resource‐constrained environments such as portable medical devices or edge systems.

To mitigate this challenge, the proposed NeuroDL framework employs transfer learning to harness pretrained CNNs, thus reducing training time by reusing robust feature extractors trained on large‐scale datasets. More importantly, by integrating lightweight ML classifiers—such as SVMs and RFs—at the decision layer, the system offloads some computational burden, effectively balancing performance and efficiency. This hybridization enables the system to avoid retraining entire networks while still achieving high diagnostic accuracy. Experimental results confirm that the proposed framework maintains an acceptable time complexity: training durations range from 7 to 18 min, depending on the CNN–ML combination used, and real‐time inference latency remains under 1.2 s per image, supporting its feasibility for clinical applications (Table 7). For further improvement, future research may explore model compression techniques such as pruning, quantization, and knowledge distillation, along with hardware acceleration through GPUs or TPUs, to enable broader scalability and deployment in diverse clinical environments (Pal et al. 2024).

**TABLE 7:** Training time and inference latency of proposed CNN–ML combinations.

CNN architecture	Classifier	Training time (min)	Inference latency (sec/image)
VGG16	XGBoost (M1)	10.2	0.85
VGG16	Random Forest (M2)	9.3	0.91
VGG16	SVM (M3)	11.0	0.79
ResNet50	XGBoost (M4)	12.5	0.88
ResNet50	Random Forest (M5)	11.8	0.93
ResNet50	SVM (M6)	14.3	0.74
InceptionV3	XGBoost (M7)	15.7	1.12
InceptionV3	Random Forest (M8)	16.1	1.18
InceptionV3	SVM (M9)	17.9	1.20

## 5 Conclusion and Future Directions

In this study, we proposed a hybrid DL and ML framework—NeuroDL—for the accurate classification of AD and BT conditions using MRI imaging data. By leveraging pretrained CNNs such as VGG16, ResNet50, and InceptionV3 for deep feature extraction and integrating them with conventional classifiers like XGBoost, SVMs, and RFs, we achieved highly reliable and interpretable diagnostic results. Among all model combinations, the ResNet50 + SVM configuration (M6) delivered the best performance, achieving an overall *F*1‐score of 0.9821, precision of 1.0, and recall of 1.0 on the Non‐Diseased class. The statistical comparison using the Wilcoxon signed‐rank test demonstrated the significant superiority of our proposed model over other state‐of‐the‐art configurations, with *p* values far below the 0.05 threshold.

The study also evaluated the computational efficiency of the proposed model. The training time ranged between 18 and 25 min, and the real‐time inference time was maintained under 1.2 s per image, making the framework suitable for clinical integration. Additionally, our comprehensive time complexity analysis revealed that the hybrid strategy not only enhances accuracy but also significantly reduces the retraining overhead compared to end‐to‐end DL models.

The key contributions of this study can be summarized as follows:

- Development of a hybrid transfer learning‐based diagnostic framework that combines deep CNN feature extraction with ML classifiers for high‐performance AD and BT detection.
- Extensive evaluation on multiple benchmark datasets, yielding state‐of‐the‐art results with statistically significant improvements over existing methods.
- Integration of time complexity analysis and runtime profiling, demonstrating the model's practical feasibility for real‐time and resource‐constrained clinical environments.
- Validation using statistical hypothesis testing to ensure the reliability and reproducibility of results across different models.

In the future, we aim to further optimize the system by applying model compression techniques, exploring multimodal inputs (e.g., PET + MRI), and leveraging hardware accelerators like GPUs/TPUs to expand the deployment of this framework across diverse healthcare infrastructures. Additionally, expanding the dataset with more demographic diversity and incorporating longitudinal data could further improve generalizability and early diagnosis capability. The integration of DL and traditional ML techniques presents a promising avenue to further enhance the accuracy, robustness, and clinical applicability of neurological disease diagnostic systems such as NeuroDL. DL models, especially CNNs, excel at automatic feature extraction from complex imaging data like MRI scans, capturing intricate patterns that may be imperceptible to human experts or traditional methods. However, DL models often require large annotated datasets and can sometimes lack interpretability, which limits their standalone clinical adoption.

By combining DL with conventional ML classifiers—such as SVMs, RFs, or ensemble learning methods—the strengths of both approaches can be synergistically leveraged. DL models can serve as powerful feature extractors that provide rich, high‐dimensional representations of input data, which are then refined and classified using ML algorithms that may offer better interpretability, robustness to smaller datasets, or enhanced generalization across diverse populations. This hybrid approach can also facilitate multimodal data integration (e.g., MRI, PET, and genetic markers), improving early disease detection and personalized treatment planning. Moreover, the complementary nature of DL and ML can address challenges such as overfitting, class imbalance, and variability in data quality. As highlighted in the reviewed literature, approaches that blend DL feature extraction with ML classification have shown improved diagnostic accuracy and reliability compared to standalone models. Future research should explore optimized architectures that dynamically combine deep feature learning with ML classifiers, incorporate attention mechanisms for interpretability, and apply transfer learning strategies to leverage diverse datasets effectively.

## Author Contributions

**Saroj Kumar Pandey**: conceptualization, investigation, writing–review and editing, writing–original draft, visualization, formal analysis, methodology. **Yogesh Kumar Rathore**: funding acquisition, writing–original draft, writing–review and editing. **Sunakshi Mehra**: methodology, validation. **Anurag Sinha**: writing–review and editing, visualization, project administration, methodology, writing–original draft, supervision. **Tarun Raj Kumar**: conceptualization. **Ankit Kumar**: visualization. **Rekh Ram Janghel**: validation. **Ayodele Lasisi**: software. **Quadri Noorulhasan Naveed**: project administration. **Md. Sazid Reza**: supervision, data curation.

## Ethics Statement

The authors have nothing to report.

## Consent

The authors have nothing to report.

## Conflicts of Interest

The authors declare no conflicts of interest.

## Peer Review

The peer review history for this article is available at https://publons.com/publon/10.1002/brb3.70788

## Data Availability

The data for this research is will be available upon request.
